# The spatial orientation of crista ampullaris: implications for BPPV diagnosis and treatment

**DOI:** 10.3389/fneur.2024.1401041

**Published:** 2024-07-04

**Authors:** Mi Zhou, Jiesheng Mao, Xiaokai Yang

**Affiliations:** ^1^Wenzhou Key Laboratory of Intelligent Medicine for Neurodegenerative Diseases, Third Affiliated Hospital, School of Medicine, Shanghai University, Shanghai, China; ^2^Wenzhou Key Laboratory of Intelligent Medicine for Neurodegenerative Diseases, Wenzhou Third Clinical Institute Affiliated to Wenzhou Medical University, Wenzhou, Zhejiang, China; ^3^Wenzhou Key Laboratory of Intelligent Medicine for Neurodegenerative Diseases, Wenzhou People's Hospital, Wenzhou, Zhejiang, China

**Keywords:** vestibular, crista ampullaris, BPPV, semicircular canal, orientation

## Abstract

**Objective:**

This study aimed to provide a comprehensive understanding of the spatial orientation of the crista ampullaris within the inner ear and its implications for the diagnosis and management of benign paroxysmal positional vertigo (BPPV).

**Methods:**

Using high-resolution MRI scans of 55 normal inner ears, 3D models of the semicircular canals were segmented. These were complemented by detailed membrane labyrinth models from micro-CT scans of human temporal bones, accessed via the Comparative Ear Bank (www.earbank.org). A statistical shape model of inner ears and eyeballs was established, and a standardized 3D spatial coordinate system was created. The horizontal plane was defined using the top of the common crus and the bottom of the eyeballs. This calibrated reference system allowed for precise quantification of crista ampullaris orientations by calculating angles between the defined crista planes and coordinate planes.

**Results:**

The plane of the ampulla and the corresponding semicircular canal plane are nearly perpendicular to each other. In the upright position, the posterior semicircular canal crista ampullaris formed an angle of 48.9° with the horizontal plane. The relative orientations of the crista ampullaris of the lateral and superior canals were also defined. Furthermore, we identified “zero-point planes” representing crista orientations perpendicular to gravity, which resulted in minimal ampullary stimulation. A 6.2° tilt to the left in the supine position resulted in the plane of the left lateral semicircular canal crista ampullaris being parallel to the direction of gravity.

**Conclusion:**

This study elucidates the precise spatial orientation of the crista ampullaris, thereby providing an anatomical basis for understanding BPPV pathophysiology and improving the accuracy of diagnostic and therapeutic maneuvers. The findings have the potential to significantly enhance the management of BPPV and other inner ear disorders.

## 1 Introduction

Benign paroxysmal positional vertigo (BPPV) is a prevalent vestibular disorder characterized by acute vertigo episodes precipitated by sudden head movements, resulting in considerable discomfort and a negative impact on an individual's quality of life. The underlying mechanism of BPPV involves the displacement of tiny calcium carbonate crystals known as otoconia from their normal position in the otolithic organs to the semicircular canals.

Extensive research has been conducted to elucidate the mechanisms of otoconia dislodgment, their flow within the semicircular canals, and their effects on the vestibular system (1). Nevertheless, despite notable advancements, the diagnosis and treatment of BPPV, particularly cupulolithiasis, remain challenging (2). This complexity arises from the intricate anatomy of the inner ear and the dynamic interplay of otoconia within its structures.

An understanding of the spatial orientation of the membranous semicircular canals and the crista ampullaris is of great importance for the elucidation of the pathophysiology of BPPV, the refinement of diagnostic tests, and the development of effective repositioning maneuvers. Unfortunately, the literature on the spatial orientation of these structures is limited (3, 4).

Obtaining accurate and complete data on the delicate gelatinous cupula covering the crista ampullaris is challenging. Clinical MRI has limitations in visualizing the detailed anatomy of the crista ampullaris, and there is a paucity of *in vivo* data. While MR microscopy and micro-CT techniques can reveal the crista ampullaris structure, the cupula in cadaveric specimens often undergoes shrinkage and deformation.

**Figure 1:**
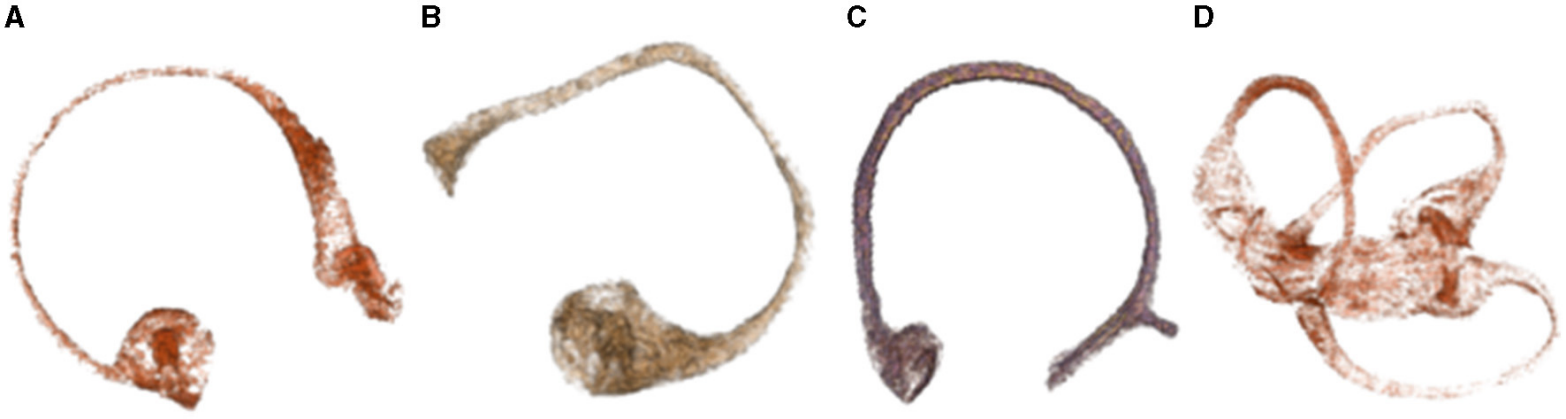
Demonstrates the structure of the crista ampullaris based on MR microscopy data. **(A)** Superior semicircular canal and crista ampullaris. The clear structure of the crista ampullaris in the ampulla can be seen at the lower left. **(B)** Lateral semicircular canal and crista ampullaris. The distinct structure of the crista ampullaris in the ampulla is visible. **(C)** Posterior semicircular canal and crista ampullaris. The structure of the crista ampullaris in the ampulla is not clearly visible. **(D)** Membranous labyrinth and crista ampullaris. The crista ampullaris of the superior and lateral semicircular canals are clearly visible.

The original image data from a study that employed MR microscopy of temporal bones after gadolinium salt immersion (5) has been analyzed. Although we were able to successfully segment and obtain membranous labyrinth models, the segmentation of the crista ampullaris structure proved to be a challenging task. The contours of the crista ampullaris in the anterior and lateral semicircular canals were relatively well-defined, but the posterior semicircular canal crista ampullaris was poorly visualized (6, 7) (Figure 1).

In our attempt to obtain temporal bone images using industrial CT, we encountered similar difficulties in clearly visualizing the cupula, which made precise spatial orientation measurements challenging.

Previous studies have sought to investigate the spatial orientation of the semicircular canals using a variety of imaging modalities. However, the majority of these studies concentrated on the bony labyrinth, with limited data on the membranous labyrinth and the crista ampullaris.

Microtomography studies by David et al. (8) provided valuable insights into the membranous labyrinth anatomy, demonstrating better visualization of the crista ampullaris. Although the cupula exhibited some shrinkage, parts of it remained intact, enabling the reconstruction of a complete crista ampullaris structure. This provides a valuable foundation for studying the spatial orientation of the crista ampullaris.

Unfortunately, the inner ear structures obtained from unilateral temporal bone scans lack inherent spatial orientation. To address this, we established a calibration method to define the spatial position of the inner ear and further investigated the spatial orientation of the crista ampullaris.

By meticulously calibrating and measuring the spatial orientation of the crista ampullaris, we seek to offer novel insights into the pathophysiology of BPPV, the challenges in diagnosis, and the development of potential therapeutic strategies. This research has the potential to contribute to the development of more precise and tailored approaches for diagnosing and treating BPPV, ultimately improving the management of this debilitating vestibular disorder.

## 2 Methods

### 2.1 MRI image data

A total of 55 patients with normal inner ears were included in the study, as determined by MRI scans conducted between January 2014 and December 2019. The study group consisted of 26 men and 29 women, with an average age of 43 years (range: 5–77 years). Inner ear examinations were conducted using a Siemens 1.5 T superconducting magnetic resonance system and a standard head coil. A three-dimensional constructive interference steady-state sequence (3D-CISS) was employed, with the following parameters: TR: 6.0 ms, TE: 2.7 ms, FOV: 135 × 18 mm, matrix: 256 × 102, and thickness: 0.7 mm.

For image processing and modeling, the original image data were exported from the Picture Archiving and Communication System (PACS) and saved in DICOM format. The images were then processed using the 3D Slicer version 4.10.2 software, with the 3D-CISS sequence automatically extracted and saved in NII format. The image spacing was 0.3515625 × 0.3515625 × 0.6999983 mm. The semicircular canals were segmented using threshold segmentation methods and smoothed using the Marching Cubes method. The utilization of these advanced imaging techniques and precise segmentation methods enabled the acquisition of accurate three-dimensional models of the semicircular canals, which are essential for the analysis of the spatial orientation of the crista ampullaris. This was accomplished in our preliminary study (9).

### 2.2 Model of the membrane labyrinth

Previous research was conducted by David et al. (8). The relevant models can be accessed via the Comparative Ear Bank website (http://www.earbank.org), which makes this study possible. Human temporal bones were obtained from donors without any known history of inner ear disorders. The specimens were fixed in Bouin solution and then stained using phosphotungstic acid (PTA) to enhance the contrast of the membranous labyrinth structures. The stained samples were scanned using a high-resolution micro-CT system (SkyScan 1173) with the following parameters: 130 kV, 61 μA, 13.57 μm isotropic voxel size, and a brass filter of 0.25 mm.

The reconstructed micro-CT images were imported into Avizo 7.1 for segmentation and 3D modeling. The membranous labyrinth structures, including the semicircular canals, ampullae, and crista ampullaris, were manually segmented slice by slice using the lasso tool in conjunction with a Cintiq 22HD screen-tablet (Wacom, Kazo, Saitama, Japan). A complete cupula without shrinkage and visualized *in-situ* corresponds to an extrusion of the stereocilia/kinocilia layer covering the corresponding crista ampullaris toward the roof of the ampulla. Extruding the shape of the crista ampullaris toward the roof of the ampulla thus provides a cupula model.

### 2.3 Spatial calibration and reference system establishment

To establish a reliable reference coordinate system for measuring the spatial orientation of the crista ampullaris, a series of spatial calibrations was performed.

The process involved multiple steps:

1) Establishment of a statistical shape model of the inner ears and eyeballs: The initial reference model was selected to be one inner ear model. The remaining models were then aligned with the reference model, and a Gaussian process model was constructed based on this reference model. Subsequently, the aligned models were further aligned point-to-point with the reference model. This iterative alignment process enabled the creation of a statistical shape model based on the aligned models.
2) Elimination of selection bias: To mitigate any potential selection bias that might have arisen from the initial choice of the reference model, we derived an average model from the statistical shape model. The average model thus became the new reference model for subsequent alignment iterations. This process was repeated until the final average model reached a stable state without significant changes. The stable average model thus constituted the standard model for calibration.
3) Establishment of a standard three-dimensional spatial coordinate system: The 3D Slicer Transform module was employed to effect a translation and rotation of the coordinate system. This alignment ensured bilateral symmetry of the semicircular canals about the sagittal plane. The horizontal plane was established by taking the top of the common crus and the bottom of the eyeballs.
4) Calibration to determine the spatial orientation of the membrane labyrinth model: The left bone labyrinth model was segmented from the micro-CT examination data and calibrated with the standard model. Additionally, a mirror image right bone labyrinth model was constructed. The calibrated bone labyrinth model served as the reference model for further modeling of the statistical shape model of the inner ears and eyeballs, allowing us to determine the spatial orientation as the standard model. Finally, the left bone labyrinth model was recalibrated with the standard model in order to transform the membrane labyrinth model into three-dimensional space, thus establishing its accurate spatial orientation.

The three-dimensional spatial coordinate system employed in this study specifies that the X-axis is positive to the right, the Y-axis is positive forward, the Z-axis is positive upward, the normal vector of the superior semicircular plane is outward, the normal vector of the posterior semicircular plane is inward, and the normal vector of the lateral semicircular plane is downward.

### 2.4 Measurement of crista ampullaris spatial orientation

Once the membranous labyrinth had been successfully calibrated, the spatial orientation of the crista ampullaris was measured with the utmost precision. This measurement process involved the following steps:

1) Determination of the plane of the crista ampullaris: The plane of the crista ampullaris was identified as a saddle-like structure within the membranous labyrinth. From this plane, we calculated the fitting plane equation, which takes the form AX + BY + CZ + D = 0.
2) Calculation of the normal vector: The normal vector of the crista ampullaris plane, represented by [A, B, C], provided valuable insights into the orientation of the crista ampullaris and its relationship to the direction of gravity in different head positions.
3) Calculations of the angle: The normal vector was employed to ascertain the angles between the crista ampullaris plane and the sagittal, coronal, and horizontal planes. These angles enabled the precise quantification of the spatial orientation of the crista ampullaris relative to the body's reference planes.

### 2.5 Crista ampullaris spatial orientation applied to BPPV diagnosis and treatment

To analyze the change in head position of the crista ampullaris in parallel or perpendicular to gravity based on the spatial orientation of the crista ampullaris, and to explore the Zero-Point Plane and the relief of the cupulolithiasis.

## 3 Results

### 3.1 Spatial orientation of the crista ampullaris

**Figure 2:**
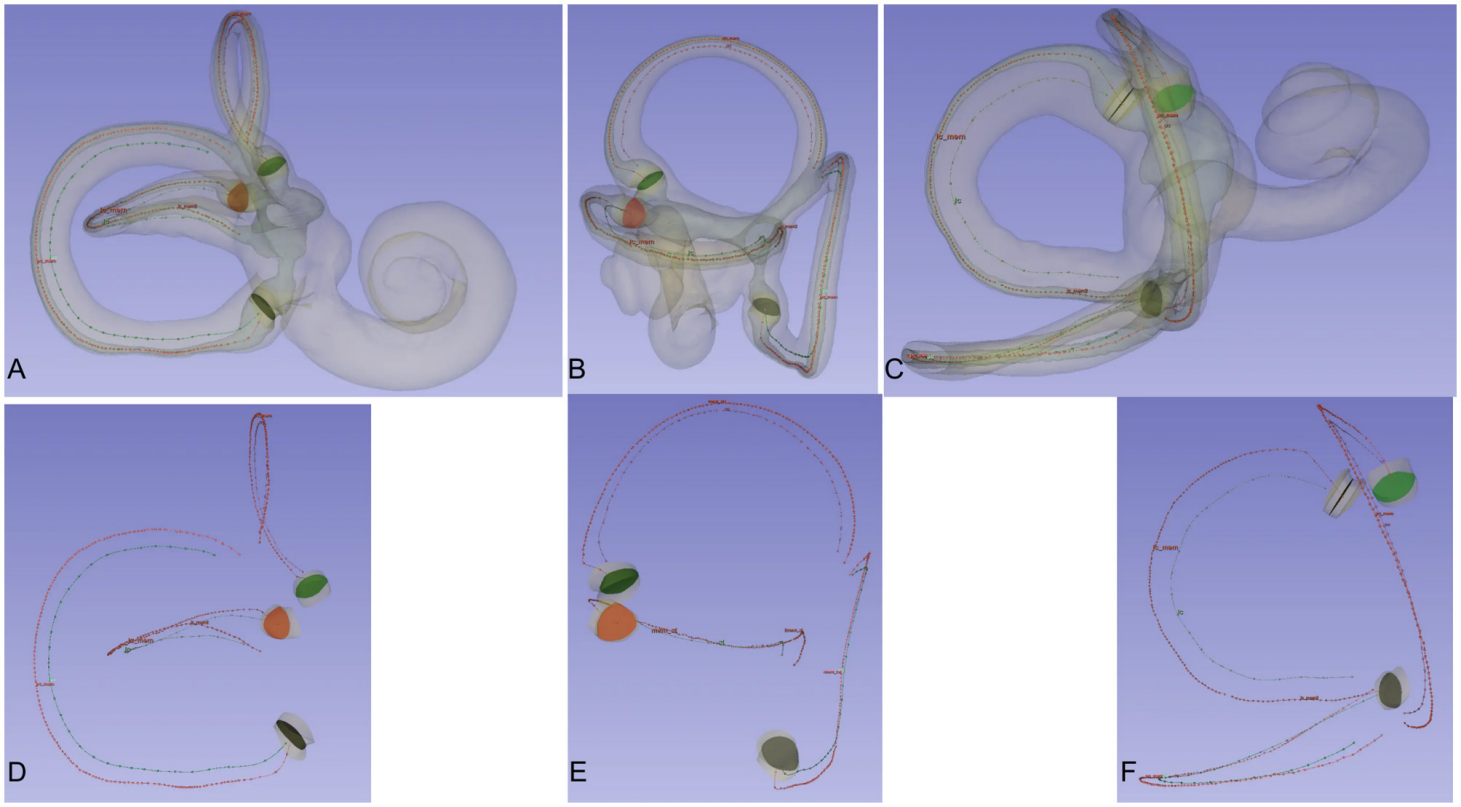
Crista ampullaris and centreline of the bony and membrane semicircular canals. Observing the spatial relationship between the central lines of the bony and membranous semicircular canals and the crista ampullaris from different perspectives. When viewing the semicircular canals from the front, the central lines of the bony and membranous semicircular canals are essentially aligned. When viewing the semicircular canals from the side, the central lines of the bony and membranous semicircular canals lie in the same plane. The crista ampullaris is perpendicular to the plane of the semicircular canals. The three semicircular canals are mutually perpendicular to each other. When viewing one semicircular canal from the front, the other two semicircular canals are positioned for side viewing. **(A)** Frontal view of the posterior semicircular canal. **(B)** View of the superior semicircular canal. **(C)** Frontal view of the lateral semicircular canal. **(D)** Frontal view of the central line of the posterior semicircular canal. **(E)** Frontal view of the central line of the superior semicircular canal. **(F)** Frontal view of the central line of the lateral semicircular canal.

The micro-CT scans and 3D reconstructions provided detailed visualizations of the membranous labyrinth and the crista ampullaris in the superior, lateral, and posterior semicircular canals (Figure 2).

Table 1 shows the spatial attitudes of different anatomical parts of the inner ear, such as the crista ampullaris, bone labyrinth, and membrane labyrinth. Table 2 provides the angles between the crista ampullaris and the respective labyrinths, as well as between the bone and membrane labyrinths, and the crista ampullaris, for reference.

**Table 1:** Inner ear spatial attitude.

**Anatomical part**	**Plane normal vector**	**Sagittal plane**	**Coronal plane**	**Horizontal plane**
LPC_CA	[−0.747 −0.1077 −0.6562]	138.33	96.18	131.01
LHC_CA	[0.9723 −0.1052 −0.2087]	13.52	96.04	102.05
LAC_CA	[0.5364 −0.0841 −0.8397]	57.56	94.82	147.11
LPC_MEM	[0.6229 −0.7016 −0.346]	51.47	134.56	110.24
LPC_BONY	[0.6553 −0.6709 −0.3472]	49.06	132.14	110.32
LHC_MEM	[−0.018 0.3302 −0.9437]	91.03	70.72	160.68
LHC_BONY	[−0.0049 0.3305 −0.9438]	90.28	70.7	160.7
LAC_MEM	[−0.7526 −0.5924 −0.2874]	138.82	126.33	106.7
LAC_BONY	[−0.7716 −0.5643 −0.2936]	140.5	124.35	107.07

LPC, left posterior semicircular canal; LHC, left lateral semicircular canal; LAC, left superior semicircular canal; CA, crista ampullaris; BN, bony labyrinth; MEM, membrane Labyrinth.

**Table 2:** Angle between the crista ampullari, the membrane, and the bony semicircular canals.

**Plane**	**Plane**	**Angle**
LPC_CA	LPC_MEM	81.93
LPC_CA	LPC_BONY	80.92
LPC_MEM	LPC_BONY	2.25
LHC_CA	LHC_MEM	80.96
LHC_CA	LHC_BONY	81.89
LHC_MEM	LHC_BONY	1.17
LAC_CA	LAC_MEM	86.11
LAC_CA	LAC_BONY	85.43
LAC_MEM	LAC_BONY	1.01
LPC_CA	LHC_CA	125.30
LPC_CA	LAC_CA	80.83
LHC_CA	LAC_CA	45.11

LPC, left posterior semicircular canal; LHC, left lateral semicircular canal; LAC, left superior semicircular canal; CA, crista ampullaris; BONY, bony labyrinth; MEM, membrane labyrinth.

The bone labyrinth and membrane labyrinth are situated in a nearly identical plane, with a difference of no more than 2.25°.

The plane of the ampulla and the corresponding semicircular canal plane are nearly perpendicular.

In the sitting position, the angle formed between the crista ampullaris and a defined horizontal reference plane was measured. Those angles varied considerably from one semicircle to another, with an angle of 131.01° for the PSC crista ampullaris, 102.05° for the LSC crista ampullaris, and 147.11° for the ASC crista ampullaris.

### 3.3 Crista ampullaris spatial orientation applied to BPPV diagnosis and treatment

**Figure 3:**
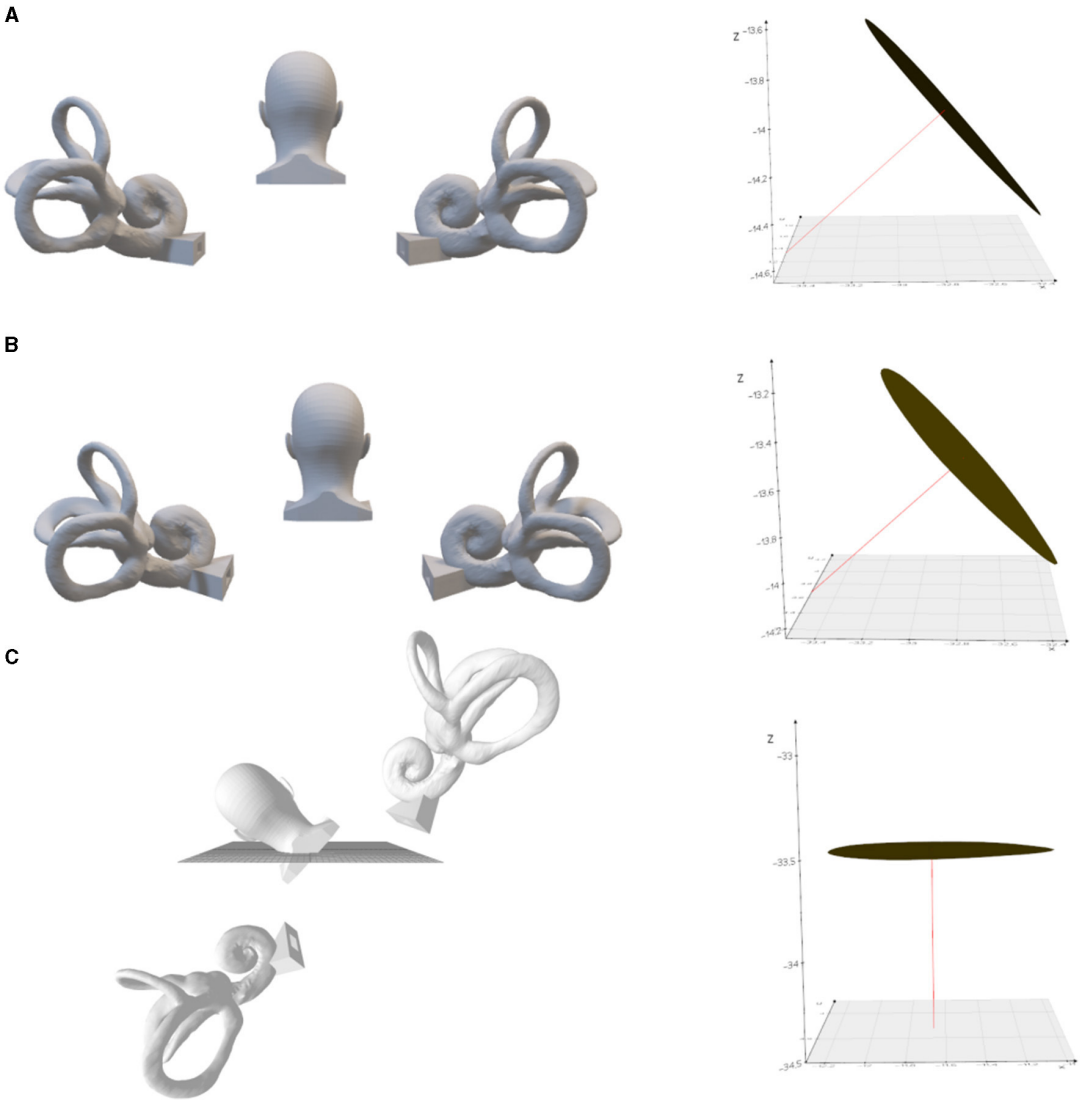
The left posterior semicircular crista ampullaris is rotated to be parallel to the horizontal plane. **(A)** Upright position. On the right is the left posterior semicircular canal crista ampullaris, where the red line is the normal vector to the plane of the crista ampullaris. **(B)** Tilted back 9.3°. **(C)** Tilted 48.3° to the left with the plane of the left posterior semicircular crista ampullaris parallel to the horizontal plane.

**Figure 4:**
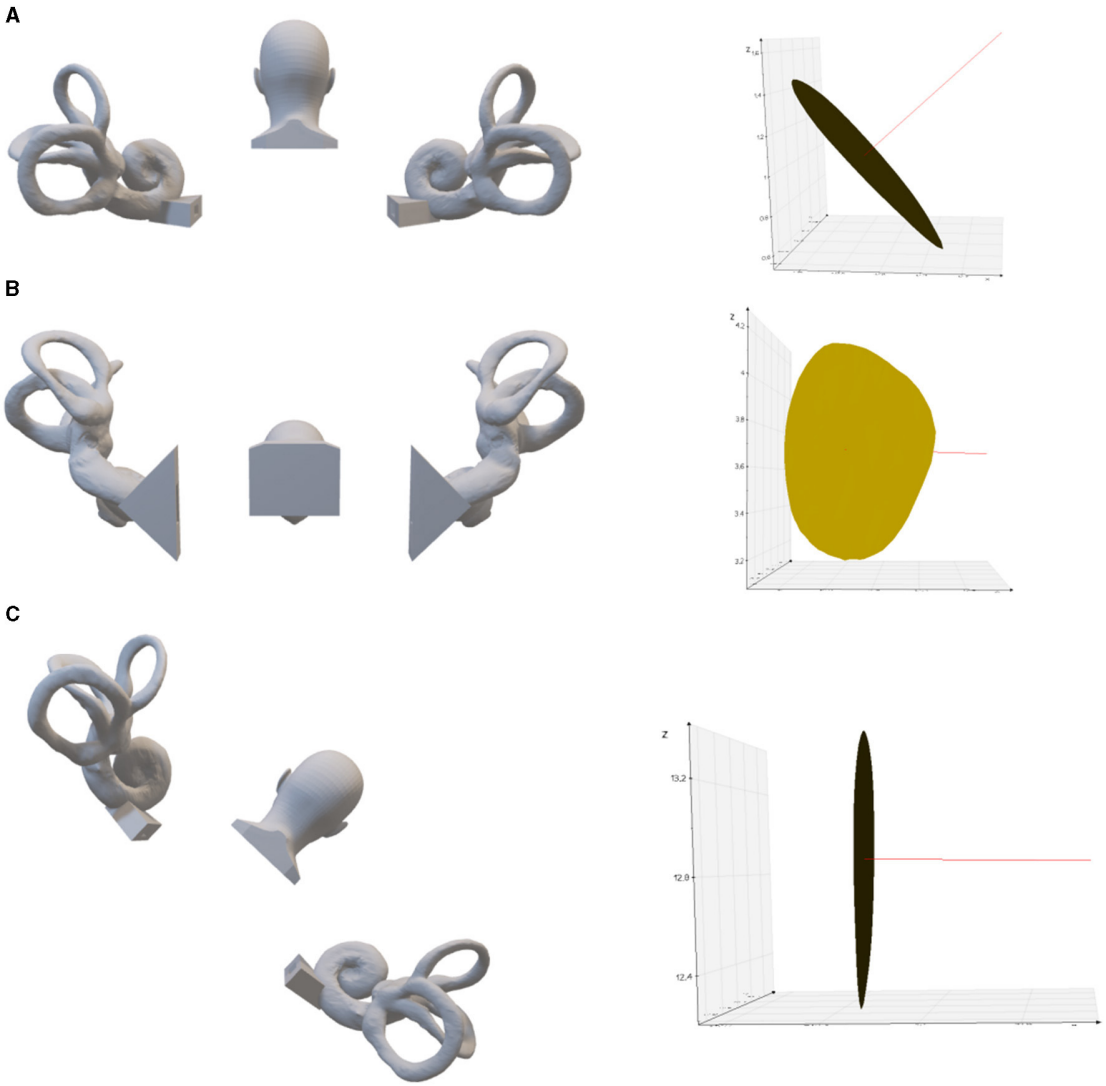
Left posterior semicircular crista ampullaris rotated to parallel the direction of gravity. **(A)** Upright position. On the right is the left posterior semicircular canal crista ampullaris, where the red line is the normal vector of the plane of the crista ampullaris. **(B)** 80.2° anterior pitch in the upright position, so that the plane of the left posterior semicircular crista ampullaris parallel to the direction of gravity. **(C)** 43.3° of lateral inclination to the right in the upright position, so that the plane of the left posterior semicircular crista ampullaris parallel to the direction of gravity.

**Figure 5:**
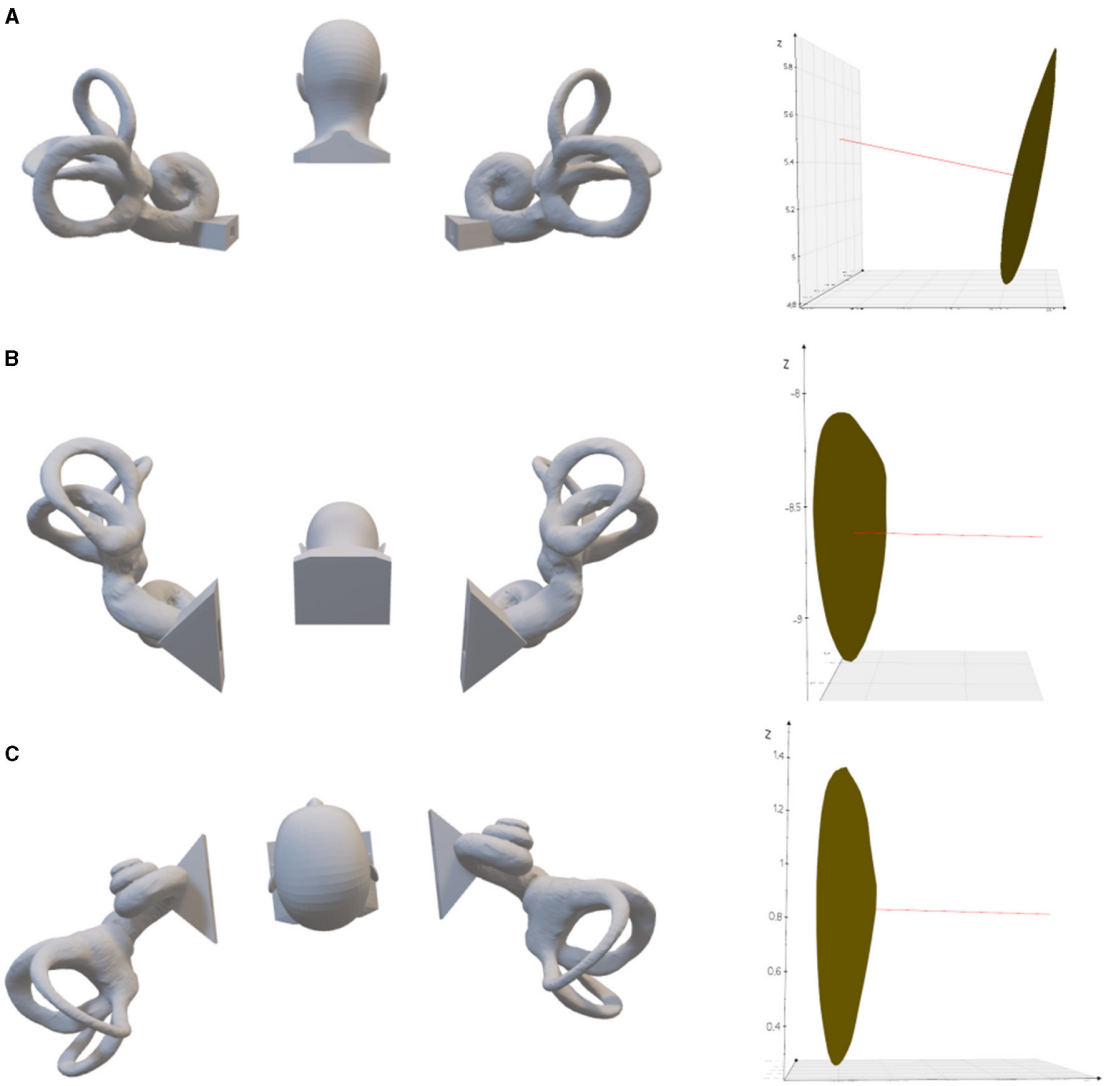
The left lateral semicircular crista ampullaris is rotated to be parallel to the direction of gravity. **(A)** Upright position. On the right is the left lateral semicircular canal crista ampullaris, where the red line is the normal vector of the plane of the crista ampullaris. **(B)** 63.22° of anterior pitch in the upright position, so that the plane of the left lateral semicircular crista ampullaris parallel to the direction of gravity. **(C)** 6.2° tilt to the left in the supine position, so that the plane of the left lateral semicircular crista ampullaris parallel to the direction of gravity.

The spatial direction of the crista ampullaris is very helpful for the diagnosis and treatment of BPPV. Based on the normal vector of the crista ampullaris plane, it can be inferred how to change the head position to make the posterior semicircular canal crista ampullaris plane perpendicular (Figure 3) or parallel to the direction of gravity (Figure 4) or to make the lateral semicircular canal crista ampullaris plane parallel to the direction of gravity (Figure 5).

## 4 Discussion

In this study, we utilized high-resolution micro-CT imaging to investigate the precise spatial orientation of the crista ampullaris within the human membranous labyrinth. The findings of this study provide a detailed quantitative analysis of the crista ampullaris angles relative to established anatomical reference planes and gravitational vectors.

The precise quantification of the crista ampullaris spatial orientation presented in this study has profound implications for furthering our understanding of the intricate biomechanical processes governing balance and vestibular function.

From a physiological standpoint, the angle of each cupula may be more crucial than that of the crista ampullaris. The cupula is offset during rotational motion, and in the quiet state, the cupula and the crista ampullaris can be assumed to be spatially oriented in the same direction.

By elucidating the complex three-dimensional anatomy of this vital structure, new opportunities have been created for enhancing the accuracy of BPPV diagnosis and optimizing therapeutic repositioning techniques tailored to the disease phenotype.

### 4.1 Significance of crista ampullaris spatial orientation

The crista ampullaris acts as a cornerstone transducer of rotational head movements via its integrated kinociliary apparatus and cupula (10, 11). The orientation of the crista ampullaris has been suggested to align with the plane of maximal stimulation from endolymph flow induced during head rotation (12–14).

There is little research data on the spatial orientation of the crista ampullaris, the biggest reason for this is that tissue section images usually lack spatial information, and although imaging technology has progressed in recent years, clinical MRI examinations cannot show the structure of the crista ampullaris, and the magnetic resonance microscopy and micro-CT are also only examined on one side of the temporal bone, and there is a similar lack of spatial information problem (3, 5, 6).

David used temporal bone staining followed by micro-CT scanning to segment the membrane labyrinth to obtain the membrane labyrinth structure, providing the first publicly available membrane labyrinth model including the crista ampullaris structure, which is of great reference significance (8). David established the spatial orientation of the membranous labyrinth by calibrating the bone labyrinth obtained by segmentation of the micro-CT scan data with a case of bone labyrinth used as a reference head, and then synchronizing the three-dimensional spatial transformation of the membranous labyrinth. Because of individual differences in the spatial orientation and shape of the semicircular canals, the reliability of their alignment and the representativeness of the reference model needs to be confirmed. In our study, we used David's inner ear model but aligned it with a standard bone semicircular canal model for a more rational approach.

Takagi et al. (15) studied the spatial relationship between the semicircular canal and the crista ampullaris by using computer three-dimensional reconstruction of tissue slices of the right temporal bone in a 14-year-old female. Select the points at both ends of the sensory epithelial cells of the ampulla crista and draw a line between them to define the long axis of the ampulla crista. The angle between the plane of the anterior semicircular canal and the long axis of the crista ampullaris is 62.9°, the angle between the plane of the lateral semicircular canal and the long axis of the crista ampullaris is 65°, and the angle between the plane of the posterior semicircular canal and the long axis of the crista ampullaris is 59.6°. There is a significant difference between Takagi's research results and ours, and the difference in measurement methods may be the main reason.

The crista ampullaris is a saddle-like structure (16), and Takagi's use of straight lines constructed at both ends of the sensory epithelium as the long axis of the crista ampullaris cannot represent the plane of the crista ampullaris. Our measurement data shows that the plane of the crista ampullaris is nearly perpendicular to the plane of the semicircular canal where it is located.

HALL believes that in the upright position, the posterior semicircular canal crista ampullaris is close to vertical, and in the Dix Hallpike induction test, it is close to horizontal (17). However, Epley proposed the Half Dix Hallpike test for the diagnosis of posterior semicircular canal cupulolithiasis, based on an angle of 60° between the posterior semicircular canal crista ampullaris and the horizontal plane in the upright position (3).

Baloh reported that the crista ampullaris of the horizontal semicircular canal in the supine position is inclined to the inner side by about 45° (18), but other studies have reported that the crista ampullaris of the lateral semicircular canal is inclined to the outer side in the supine position (19, 20), with significant inconsistency.

In the present study, the measurements were 41.7° between the crista ampullaris of the posterior semicircular canal and the sagittal plane in the upright position and 48.9° angle with the horizontal plane, which is close to Epley's opinion.

The angle between the crista ampullaris of the lateral semicircular canal and the sagittal plane in the lying position was 13.52°, which was skewed toward the side of the utricle.

Our study provides quantitative evidence substantiating this hypothesis through the direct measurement of crista angles relative to the semicircular canal planes. By defining the precise resting state orientation, our data permits clarifying the head motion vectors eliciting maximal depolarization of each ampullary nerve and additionally explains the variable nystagmus patterns associated with differential canalithiasis. Furthermore, by calculating the normal vectors, we demonstrate an ingenious method to derive the crista angles dynamically for any head position. This sets the stage for unprecedented precision in maneuvering patients into optimal orientations for liberating trapped otoconia.

### 4.2 The “Zero-Point Plane” concept

A particularly salient finding of our study was the calculation of the “zero-point plane” for each crista—the orientation perpendicular to gravity in which there is no background stimulation of the crista. This has profound diagnostic utility, as otoconial debris on an orthogonally positioned crista produces negligible vertigo or nystagmus (21), thereby distinguishing it from canalolithiasis (22). Hence, identifying the specific plane in which nystagmus or vertigo is absent can precisely pinpoint the affected side and semicircular canal harboring the pathogenic otoconia. Additionally, applying simple trigonometric principles to our measured normals enables facile identification of the zero-point plane for customized diagnostic positioning per patient. Our data therefore heralds the advent of a new paradigm in BPPV diagnosis leveraging the zero-point concept (23).

Our findings suggest that a 6.2° tilt to the left in the supine position aligns the plane of the left lateral semicircular crista ampullaris parallel to the direction of gravity, defining the “Zero-Point Plane.” However, this angle is notably smaller than the reported neutral position angles for direction-changing positional nystagmus in BPPV patients, which average around 26.5 ± 11.6° (24).

Several factors may contribute to this discrepancy. Firstly, it is important to distinguish between the crista ampullaris plane and the Zero-Point Plane. Although related, they may not be precisely the same. Bisdorff et al. objectively reported the Zero-Point Plane angles in BPPV patients with lateral canal cupulolithiasis, finding that the Zero-Point Plane is reached when the head is turned 10–20° to the affected side in the supine position (4). However, their study did not directly investigate the relationship between the Zero-Point Plane and the crista ampullaris plane.

Secondly, the three-dimensional geometry of the crista ampullaris, particularly its saddle-like shape (16), may influence the Zero-Point Plane angle. Even when the crista ampullaris plane is parallel to gravity, otoconia adhered to the crista of this structure may still exert a pulling effect, resulting in a larger Zero-Point Plane angle.

Thirdly, individual differences in the location of otoconia could contribute to the variability observed in neutral point angles. From an anatomical standpoint, the crista ampullaris plane angles should not exhibit such substantial variability, although some individual differences may exist.

### 4.3 Strategies for optimized otoconial repositioning

The zero-point plane further exhibits therapeutic relevance, as aligning the affected crista perpendicular to gravity predisposes for otoconial detachment back into the endolymph due to minimized background firing (25, 26). Tilting the head to orient the crista parallel to gravity is even more effective, as this equally promotes the dislodgement of otoconia on either side of the crest (27, 28). Our study provides the mathematical framework essential for exploiting this strategy via the 3D coordinates we defined. By aligning the patient so the culminant vector between the affected crista and gravity is minimized, otoconial clearance can be enhanced. This may be supplemented by maneuvers strategically incorporating rotational head shaking (29) along the axis of the canal pair bracketing the symptomatic crista to augment endolymphatic flow. Devising therapy protocols rationally guided by the crista angles derived from our methodology therefore holds immense promise for optimizing BPPV repositioning outcomes.

### 4.4 Therapeutic implications and future directions

The use of magnetic fields or ultrasound to stimulate the crista ampullaris is an effective method for achieving precise vestibular stimulation in the future. The study of the spatial posture of the crista ampullaris provides an important anatomical basis for the stimulation of the crista ampullaris by magnetic field or ultrasound.

### 4.5 Limitations of the study

In this study of the spatial orientation of the crista ampullaris, only one case of data provided by David et al. was used. The primary reason for this limitation is the current technological challenges in acquiring large-scale data on the membranous labyrinth, particularly the intricate structure of the crista ampullaris. As discussed earlier, clinical MRI has limitations in visualizing these delicate structures, and while MR microscopy and micro-CT techniques offer better visualization, they are often performed on cadaveric specimens, which may introduce deformations due to tissue shrinkage and preservation methods.

We acknowledge that relying on a single specimen may not fully capture the extent of individual variability in the anatomy of the membranous labyrinth and the crista ampullaris. To date, there have been few studies that have successfully acquired high-resolution data on the membranous labyrinth and crista ampullaris from a large number of human specimens. The complex and delicate nature of these structures, combined with the need for specialized imaging techniques and tissue preparation methods, has hindered the large-scale acquisition of such data.

Despite this limitation, our study offers valuable insights into the spatial orientation of the crista ampullaris, providing a clearer understanding of its anatomical position. While individual variations may exist, they are likely to be limited in scope.

Future research should aim to clarify the precise relationship between the crista ampullaris plane and the Zero-Point Plane, taking into account factors such as the three-dimensional geometry of the crista ampullaris and the location of otoconia. A better understanding of these aspects will provide valuable context for interpreting existing literature and guide the development of more targeted diagnostic and therapeutic approaches for BPPV.

## 5 Conclusion

In summary, our research constitutes the seminal application of high-resolution 3D modeling and vector mathematics to quantifying crista ampullaris biomechanics. By laying this robust methodological foundation, we have paved the way for an exciting new era of bespoke BPPV diagnostics and therapeutics designed around the patient's unique crista orientation. Our findings herald a future of precision otological medicine optimized for the requirements of each individual's vestibule-ocular apparatus.

## Data availability statement

The original contributions presented in the study are included in the article/supplementary material, further inquiries can be directed to the corresponding author.

## Ethics statement

The studies involving human participants were reviewed and approved by the Ethics Committee of Wenzhou People's Hospital. Written informed consent from the patients/participants or patients/participants' legal guardian/next of kin was not required to participate in this study in accordance with the national legislation and the institutional requirements.

## Author contributions

MZ: Writing – original draft, Visualization, Validation, Data curation. JM: Writing – review & editing, Validation, Software, Data curation. XY: Writing – review & editing, Writing – original draft, Software, Project administration, Methodology, Funding acquisition, Conceptualization.
